# All Properties of Infertility Microbiome in a Review Article

**DOI:** 10.1002/jcla.25158

**Published:** 2025-03-09

**Authors:** Zahra Elahi, Maryam Mokhtaryan, Shiva Mahmoodi, Soheila Shahroodian, Taleih Darbandi, Fatemeh Ghasemi, Roya Ghanavati, Atieh Darbandi

**Affiliations:** ^1^ Department of Microbiology, School of Medicine Iran University of Medical Sciences Tehran Iran; ^2^ Vice Chancellery of Education and Research Torbat Heydariyeh University of Medical Sciences Torbat Heydariyeh Iran; ^3^ Departman of Internal Medicine Shiraz University of Medical Sciences Shiraz Iran; ^4^ School of Medicine Kermanshah University of Medical Sciences Kermanshah Iran; ^5^ Department of Pharmacy, Tehran Medical Sciences Islamic Azad University Tehran Iran; ^6^ Medical Microbiology Research Center Qazvin University of Medical science Qazvin Iran; ^7^ Behbahan Faculty of Medical Sciences Bhabhan Iran; ^8^ Molecular Microbiology Research Center Shahed University Tehran Iran

**Keywords:** dysbiosis, infertility, microbiome, reproductive health

## Abstract

**Background:**

The microbiome is crucial for many physiological processes, including immunity, metabolism, and reproduction.

**Aims:**

This review aims to contribute to a detailed understanding of the microbiome of the genital tract, which can lead to better management of dysbiosis and reproductive disorders.

**Methods:**

Data from the four international information databases Medline, Scopus, Embase, and Google Scholar. The search strategy was based on the combination of the following terms: “microbiota,” “microbiome,” “microfilm,” “microflora,” “fertility,” or “infertility.”

**Result:**

The advent of next‐generation sequencing‐based technologies during the last decade has revealed the presence of microbial communities in nearly every part of the human body, including the reproductive system. Several studies have shown significant differences between the microbiota of the vagina and endometrium, as well as other parts of the upper genital tract.

**Discussion:**

The human microbiome plays a critical role in determining a person's health state, and the microbiome of the genital tract may impact fertility potential before and after assisted reproductive treatments (ARTs).

**Conclusion:**

To completely understand the role of the microbiome, future research should focus not only on the description of microbiota but also on the interaction between bacteria, the production of biofilms, and the interaction of microorganisms with human cells.

## Graphical abstract

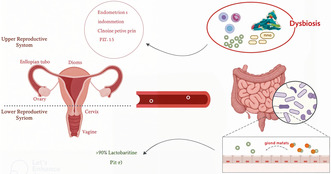

**Abbreviations**
A. vaginae: Atopobium vaginae
BMI: body mass index
BV: Bacterial vaginosis
C. albicans: Candida albicans
C. trachomatis: Chlamydia trachomatis
E. coli: Escherichia coli
EPSs: exopolysaccharides
FMT: Fecal Microbiota Transplantation
G. vaginalis: Gardnerella vaginalis
HPV: Human papillomavirus
IVF: in vitro fertilization
L. jensenii: Lactobacillus jensenii
L. crispatus: Lactobacillus crispatus
M. genitalium: Mycoplasma genitalium
BMI: Mass index
MUFA: Monounsaturated fatty acids
NG: Neisseria gonorrhoeae
NGS: next‐generation sequencing
PID: pelvic inflammatory disease
PCOS: polycystic ovary syndrome
P. gingivalis: Porphyromonas gingivalis
PUFA: Polyunsaturated fatty acids
P. aeruginosa: Pseudomonas aeruginosa
qPCR: quantitative PCR
RIF: Recurrent implantation failure
ART: Reproductive technologies
S.agalactiae: Streptococcus agalactiae
S.aureus: Staphylococcus aureus
S. sanguinis: Streptococcus sanguinis
S. mitis: Streptococcus mitis
STIs: sexually transmitted infections
SFBs: Segmented filamentous bacteria
T. denticola: Treponema denticola
T. forsythia: Tannerella forsythia
U. urealyticum: Ureaplasma urealyticum
VMT: Vaginal Microbiota Transplant trials

## 1 Introduction

The American Society for Reproductive Medicine (ASRM, 2023) has described infertility as a medical condition that involves the inability to achieve a successful pregnancy. This can be due to various factors such as medical history, sexual history, reproductive history, age, physical examinations, diagnostic tests, or a combination of these elements. Infertility may require medical intervention, which could include the utilization of donor gametes or embryos to facilitate a successful pregnancy, whether the individual is trying to conceive alone or with a partner. In cases where there is no apparent cause of infertility despite regular unprotected intercourse, evaluation should commence after 12 months for female partners under 35 years of age and after 6 months for female partners aged 35 years or older [1]. Infertility affects almost one in six people worldwide at some point in their lives [2]. The prevalence of infertility among couples of reproductive ages is between 12.6% and 17.5% worldwide [3].

The term “microbiome” describes the assortment of bacteria, fungi, and viruses that inhabit and are present in and on the human body [4]. The microbiome is crucial for many physiological processes, including immunity, metabolism, and even reproduction [5]. An imbalance in the microbiome, referred to as dysbiosis, could be a factor in the inability to conceive for both males and females [6]. Several reproductive disorders, such as pelvic inflammatory disease and endometriosis, have been associated with an imbalance in the vaginal microbiome of women [7]. Also, studies showed that lower quality of sperm and infertility have been linked to imbalances in the gut microbiome in men [8]. So, various factors such as race, age, habits, and sexual activity can play a role in the diversity of microbiota in the genital region [9]. The connection between microbial origins of pelvic inflammatory disease (PID) leading to infertility is associated with sexually transmitted microorganisms, such as *Chlamydia trachomatis* ( *C*. *trachomatis* ), *Neisseria gonorrhoeae* , *Mycoplasma genitalium* ( *M*. *genitalium* ), and microorganisms linked to bacterial vaginosis, mostly anaerobic [10].

*M*. *genitalium* is a type of bacteria that can cause infertility and is transmitted through sexual contact. Research indicates that women who have *M*. *genitalium* infections have a higher likelihood of experiencing infertility due to damage or dysfunction of the fallopian tubes [11, 12]. Furthermore, *Gardnerella vaginalis* (*G*. *vaginalis*), a bacterium frequently linked with bacterial vaginosis, has been connected to female infertility as it can disturb the balance of the vaginal microbiome and trigger inflammation [13]. Prostatitis and epididymitis, which may result in poor sperm quality and function and ultimately infertility, have been linked to *Escherichia coli* ( *E*. *coli* ) and other uropathogenic bacteria in males [14]. *M*. *genitalium* is another bacterium that has been linked to male infertility, and it can cause urethritis, prostatitis, and epididymitis. Additionally, this bacterium can result in reduced sperm motility and changes in sperm morphology [15]. *Lactobacillus* has been suggested as a potential probiotic for maintaining semen quality and could also be beneficial in mitigating the harmful effects of *Prevotella* and *Pseudomonas* [8]. Adjusting the microbiome equilibrium using alterations in diet, probiotics, or transferring fecal microbiota may enhance fertility results for males and females [16].

The purpose of this review is to collect information about the microbiome of infertility in women and men and investigate how the type of microbiome affects future fertility.

## 2 Material and Methods

Data were from the four international information databases Medline, Scopus, Embase, and Google Scholar. The search strategy was based on the combination of the following terms: “microbiota,” “microbiome,” “microfilm,” “microflora,” “fertility,” or “infertility.” Each keyword combination was linked using the Boolean operators OR and AND. The search strategy was adapted to the specifications of each database. We evaluated the selected studies by examining their titles, abstracts, and full texts. To filter out irrelevant studies, a set of exclusion criteria was applied, such as modeling studies, commentaries, duplicate articles, editorials, guidelines, news articles, and studies lacking adequate data. Finally, duplicate articles were identified and removed using EndNote X9 software (Thomson Reuters, San Francisco, CA).

## 3 Microbiota in the Female Reproductive System

### 3.1 The Relationship Between the Microbiome and Infertility

**FIGURE 1:**
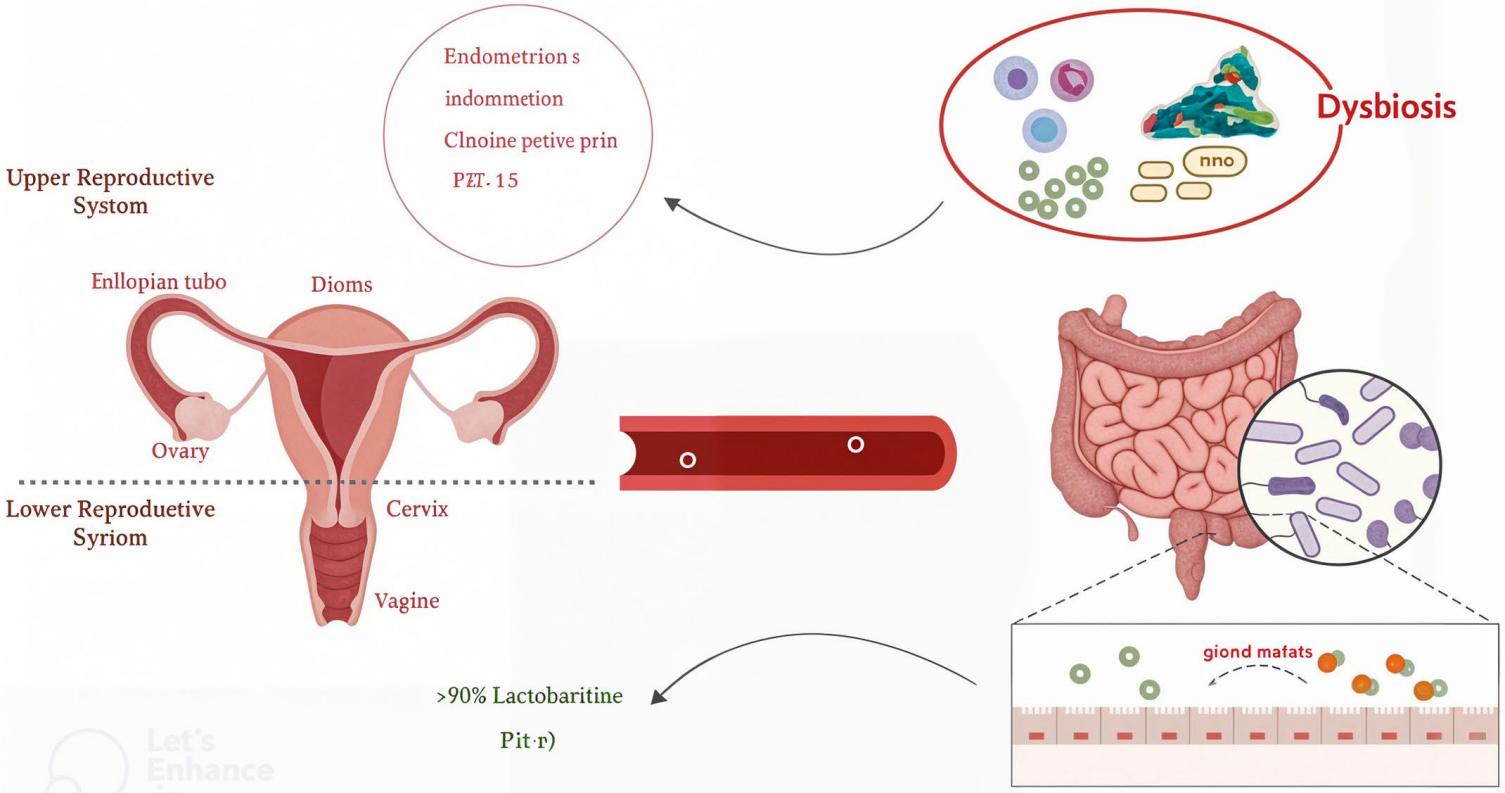
Schematic representation of the effect of the gut microbiome on modulating or destroying the female reproductive system through changing estrogen levels.

The female genital tract microbiome is a crucial determinant of women's reproductive health. A harmonious vaginal microbiome, dominated by *Lactobacillus* species, creates an acidic vaginal environment that inhibits the proliferation of pathogenic microorganisms, thereby conferring protection against infections. Alterations in this intricate microbial balance, termed dysbiosis, can precipitate bacterial vaginosis, sexually transmitted infections, and other reproductive health disorders [17]. In recent years, the potential impact of the microbiome on infertility in women has garnered increased attention [18, 19, 20]. Imbalances in the vaginal microbiome have been implicated in various gynecological conditions that can affect fertility, such as polycystic ovary syndrome (PCOS), endometriosis, pelvic inflammatory disease as well as cervical cancer and its specific treatment [6, 21]. Furthermore, investigations have established correlations between distinct microbial profiles and in vitro fertilization (IVF) outcomes. For example, women with a reduced prevalence of *Lactobacillus* species in their vaginal microbiome have exhibited diminished probabilities of successful embryo implantation and live birth after undergoing IVF treatment [22]. Considering the burgeoning body of research evidence implicating the microbiome in reproductive health and fertility, there is significant scientific and clinical merit in comprehensively evaluating and understanding the microbiome to optimize fertility outcomes. Through the comprehensive characterization of the vaginal microbiome in women experiencing infertility, healthcare providers can precisely identify potential microbial imbalances and prescribe tailored interventions, such as probiotics or antibiotics, to restore a healthy microbial equilibrium [23]. Furthermore, understanding the role of the microbiome in fertility can help inform the development of novel therapeutic strategies, such as microbiome‐based diagnostics or treatments, that could improve the success rates of assisted reproductive technologies like IVF [24]. In conclusion, the microbiome inhabiting the female genital tract exerts a profound influence on women's reproductive health and fertility (Figure 1). Persistent scientific inquiry into the microbiome and its contributions to fertility may culminate in novel diagnostic and therapeutic approaches that can enhance the chances of conception for women encountering difficulties in achieving pregnancy [25].

### 3.2 Vaginal Microbiome and Infertility

The role of the vaginal microbiome in female reproductive health has become an area of increasing interest in recent years [19]. Studies have demonstrated that the vaginal microbiome plays a critical role in maintaining a healthy reproductive tract and that disturbances in the microbial balance can contribute to the development of gynecological conditions, including infertility [26]. For instance, alterations in the vaginal microbiota can trigger an inflammatory response and inflict harm on the reproductive organs, impeding the process of fertilization. Moreover, an imbalance in the vaginal microbiome, characterized by the overgrowth of pathogenic bacteria, can provoke heightened production of reactive oxygen species, which can impair sperm viability and curtail their motility [27]. Dysbiosis of the vaginal microbiota, marked by an imbalance in the microbial composition, has been linked to multiple gynecological disorders, such as infertility. Several studies have explored the prevalence of vaginal dysbiosis in women suffering from infertility [28]. As an illustration, a particular study reported that women affected by unexplained infertility exhibited a higher incidence of vaginal dysbiosis, with an overabundance of pathogenic bacteria, when compared to women with normal fertility [29]. In another study, it was observed that women with a history of infertility had reduced levels of beneficial *lactobacilli* in their vaginal microbiome compared to women with normal fertility. These results indicate that vaginal dysbiosis may represent a shared etiological factor in infertility and that rectifying the microbial imbalance could be a crucial facet of infertility management [27]. While the role of seminal *Lactobacillus* remains disputed, vaginal *Lactobacilli* have long been regarded as entirely beneficial components of the genital system. The adhesion of vaginal *Lactobacillus* to spermatozoa may differ from that of seminal *Lactobacillus* at high concentrations [30]. Spermatozoa, once ejaculated, are motile planktonic cells. As spermatozoa motility is a crucial factor in assessing fertility, bacterial adhesion may increase the load on spermatozoa, consequently reducing their motility. Spermatozoa with lower motility have a higher probability of adhering, and bacterial binding increases their burden, resulting in decreased motility. If there are significant concentrations of bacteria in the vaginal canal, these spermatozoa may be deposited and lose their viability. Bacteria can cause harm if they attach themselves to spermatozoa at specific locations. Bacteria adhering to the spermatozoa's acrosome can potentially obstruct vital processes involved in fertilization. Therefore, bacteria that exhibit a strong inclination toward attaching to the acrosome may pose a significant threat. Immobilized spermatozoa can serve as a nucleus, attracting planktonic bacteria to form complexes [31]. Upon reaching a certain concentration of planktonic bacteria, numerous complexes can agglutinate and form an enormous net structure that might impede the normal swimming of spermatozoa. Widespread bacterial agglutination can trigger the secretion of exopolysaccharides (EPSs) and initiate the production of biofilms. Additionally, certain exotoxins have the potential to immobilize spermatozoa and hinder their capacity to fertilize, even when they are directly attached. These harmful consequences require high bacterial concentrations, which are uncommon in semen but frequent in the vaginal canal [32]. Numerous investigations have undertaken a comparative evaluation of the vaginal microbiome in healthy women and women afflicted with infertility. Most of these studies have reported that women with infertility exhibit a less diverse and less stable vaginal microbiota in contrast to healthy women, characterized by an augmented abundance of pathogenic bacteria and a reduced prevalence of beneficial *lactobacilli* [33]. For example, one study found that infertile women had a higher abundance of *G*. *vaginalis* and *Atopobium vaginae* ( *A*. *vaginae* ), two bacteria associated with vaginal dysbiosis, compared to fertile women [34]. Sialidase generated by *G*. *vaginalis* may accelerate the entry and proliferation of papilloma and other sexually transmitted viruses, according to data from in vitro investigations. Furthermore, it can be argued that *G*. *vaginalis* and bacterial vaginosis are associated with the development of uterine cancer based on earlier research. However, further study is needed to fully understand the precise mechanism by which *G*. *vaginalis* and human papillomavirus (HPV) contribute to infertility [35]. Several bacterial species have been recognized as putative agents that contribute to vaginal dysbiosis and infertility in females [36]. For example, *G*. *vaginalis* is a pathogenic bacterium that is commonly associated with bacterial vaginosis, a condition linked to infertility [37]. *G*. *vaginae* has also been identified as a potential contributor to vaginal dysbiosis and infertility, with a higher prevalence in women with unexplained infertility compared to fertile women [26]. Additional bacterial taxa, including *Prevotella* spp. and *Mobiluncus* spp., have been implicated in the pathogenesis of vaginal dysbiosis and infertility. Conversely, *lactobacilli* represent beneficial members of the vaginal microbiota, and their diminished prevalence has been associated with infertility [38, 39]. Recent studies have explored the potential of vaginal microbiome analysis as a diagnostic tool for infertility. One study found that performing a vaginal microbiome analysis can help identify women at risk of implantation failure during treatment by IVF [40, 41]. An additional investigation revealed that incorporating probiotic supplementation into IVF therapy can increase the probability of achieving pregnancy in women who have previously encountered implantation failure [42]. These findings imply that the analysis of the vaginal microbiome could represent a valuable strategy for enhancing the outcomes of infertility treatment and that probiotics may offer a promising intervention [43].

### 3.3 Uterine Microbiome and Infertility

Recent research has shown that the uterine microbiome, like the vaginal microbiome, can play a significant role in female reproductive health and infertility. The uterine microbiome is thought to be involved in the implantation and maintenance of pregnancy, with disruptions to the microbial balance potentially leading to infertility [44, 45]. The composition of the uterine microbiome is influenced by a variety of factors, including hormonal fluctuations, vaginal microflora, and the immune system [44]. New scientific research has revealed that the uterine microbiome of infertile women differs from that of fertile women. Specifically, the uterine microbiome of infertile women has lower levels of helpful bacteria and higher levels of harmful bacteria [46]. In addition, lower levels of bacterial diversity and an imbalance in the Firmicutes/Bacteroidetes ratio in the rectal area, as well as lower levels of *lactobacilli* in the endometrial microbiome, have been linked to recurrent implantation failure (RIF) in women who are experiencing infertility [42, 47]. These observations propose that preserving a healthy microbial balance of the uterine microbiota could represent a crucial determinant of fertility in women [45]. Although studies examining the uterine microbiome are in their infancy, multiple investigations have reported that perturbations in the uterine microbial community may constitute a contributory factor to infertility in females [45]. One study found that women with unexplained infertility had a higher prevalence of pathogenic bacteria in their uterine cavity compared to fertile women [48]. Several bacterial species have been recognized as potential agents that contribute to uterine dysbiosis and infertility in females [45]. For example, *G*. *vaginalis* and *Streptococcus agalactiae* (*S*. *agalactiae*) are two bacteria commonly associated with bacterial vaginosis and can also be found in the uterine microbiome [49, 50]. Other bacteria, such as *E*. *coli* and *Staphylococcus aureus* (*S*. *aureus*), have also been linked to uterine dysbiosis and infertility [44, 45]. It has been demonstrated that the *S*. *aureus* strain can adhere to human spermatozoa and impair sperm mobility by causing sperm agglutination [51]. *Escherichia coli* is the most frequently isolated pathogen from the urine and seminal fluid of patients with reproductive tract diseases, as it is the most common bacterial species responsible for infections in the male reproductive system. Similar to many other bacteria, it is hypothesized that the presence of *E*. *coli* may reduce sperm concentration, motility, and viability [52]. Conversely, *Lactobacillus* species represent beneficial members of the uterine microbiota, and their reduced prevalence has been associated with infertility [41]. The results indicate that a disrupted uterine microbiota could be a prevalent predisposing element in infertility, and rectifying the microbial dysbiosis may constitute a crucial component of infertility management [42, 53].

### 3.4 Cervicovaginal Microbiome and Infertility

The cervicovaginal microbiome plays a crucial role in maintaining vaginal health by regulating the pH level, producing antimicrobial substances, and preventing pathogenic microorganisms from colonizing the vaginal tract [26]. Several investigations have investigated the correlation between the microbiota inhabiting the cervicovaginal region and female infertility. In a study conducted by Moreno et al. in 2019, the authors found that women with infertility had a significantly different cervicovaginal microbiome composition compared to fertile women. They observed a decrease in beneficial *Lactobacillus* species and an increase in pathogenic bacteria, such as *G*. *vaginalis* , in infertile women [48, 54]. Reduced *Lactobacillus* abundance is correlated with decreased lactic acid generation, and the metabolic byproducts of anaerobic bacteria raise the normal vaginal pH, creating an advantageous niche for opportunistic infections [47]. Similarly, another study by Munoz, A in 2020 revealed that the cervicovaginal microbiome of infertile women was characterized by a higher abundance of anaerobic bacteria and a lower abundance of *Lactobacillus* spp [55]. Moreover, the authors found that the abundance of specific bacterial taxa was associated with infertility‐related factors, such as age, body mass index and ovarian reserve [48]. Cervicovaginal dysbiosis refers to an imbalance in the microbial composition of the cervicovaginal ecosystem, which can lead to an increased risk of various gynecological conditions, including infertility [56]. A systematic review and meta‐analysis by Chen et al. (2020) aimed to evaluate the prevalence of cervicovaginal dysbiosis in women with infertility. The authors analyzed 23 studies and found that women with infertility had a higher prevalence of cervicovaginal dysbiosis compared to fertile women. Furthermore, the authors observed that the prevalence of cervicovaginal dysbiosis was higher in infertile women with certain conditions, such as polycystic ovary syndrome and endometriosis [42, 57]. Comparative analyses of the upper genital tract microbiomes of fertile and infertile females have identified putative microbial signatures of reproductive dysfunction [56]. A study by Tao et al. [58] analyzed the cervicovaginal microbiome of women with unexplained infertility and found that the diversity and richness of bacterial species were significantly lower in infertile women compared to fertile women. The authors also observed a higher abundance of pathogenic bacteria, such as *Prevotella* and *Gardnerella*, in infertile women. They further found that the relative abundance of *Lactobacillus crispatus* ( *L*. *crispatus* ) was significantly lower in infertile women, while the abundance of pathogenic bacteria, such as *E*. *coli* and *S*. *agalactiae* , was higher [59, 60]. The microbiota inhabiting the cervicovaginal region comprises a varied array of bacterial taxa, with *Lactobacillus* species representing the prevailing members in women with normal microbiota [59]. Several studies have investigated the relationship between specific bacterial taxa and infertility in women. A higher abundance of *Lactobacillus iners* (*L*. *iners*) was associated with a decreased risk of infertility in women with polycystic ovary syndrome. In contrast, a higher abundance of *G*. *vaginalis* was associated with an increased risk of infertility in the same population [61, 62]. Additionally, a study conducted by Huang and Pu [63] explored the correlation between the cervicovaginal microbiota and outcomes of assisted reproductive technologies (ART) in women afflicted with infertility. The investigators established that a heightened prevalence of *L*. *crispatus* and *Lactobacillus jensenii* ( *L*. *jensenii* ) was positively linked with favorable ART outcomes. In contrast, an augmented abundance of *G*. *vaginalis* and *A*. *vaginae* was negatively associated with the outcomes of ART [64, 65].

### 3.5 Endometrium Microbiome and Infertility

The human endometrial microbiome, a complex ecosystem of microorganisms inhabiting the uterine lining, has recently emerged as a significant factor influencing female reproductive health, particularly infertility [66, 67]. Multiple investigations have revealed that modifications in the endometrial microbial composition are linked to diverse reproductive disorders, such as endometriosis, PCOS, and recurrent pregnancy loss, that can result in infertility in females [68, 69]. Consequently, the exploration and understanding of the endometrial microbiome are crucial for developing novel diagnostic and therapeutic approaches in the management of female infertility [70, 71]. The endometrial microbiome consists of diverse bacterial species, with *Lactobacillus* being the predominant genus in healthy women [72, 73]. However, a shift in the microbial balance, resulting in the overgrowth of potentially pathogenic bacteria such as *Gardnerella*, *Prevotella*, *Streptococcus*, and *Staphylococcus*, has been observed in infertile women [70, 74]. This disruption of the endometrial microbial balance may constitute a contributory factor to an adverse uterine milieu, leading to impaired implantation and embryo development [70]. Consequently, elucidating the interactions between distinct bacterial entities within the endometrial microbiome and female fecundity is critical for identifying putative signatures of pathogenesis, as well as developing focused probiotic manipulation strategies to redress microbial dysbiosis and optimize reproductive success [75]. Via molecular methods, investigators have demarcated a discrete microbial assemblage within the endometrium, manifesting divergences from the vaginal microbiota [76]. Among the identified taxa, *Lactobacillus* spp. is the most abundant operational taxonomic unit in the endometrium. However, further microbial taxa, including those that contribute to dysbiosis, have also been identified in both the endometrium and vagina. These findings suggest that the bacterial species identified may constitute the indigenous endometrial microbiota [44]. Interestingly, differences between the endometrial and vaginal flora have been observed irrespective of the method of endometrial sample collection. This confirms the existence of an indigenous endometrial microbiota, which may have important implications for reproductive health [44]. In 2016, Franasiak et al. were the first to investigate the potential impact of the endometrial microbiota on reproductive outcomes. They used 16S rRNA sequencing to analyze the microbiota at the tip of the transfer catheter in 33 patients undergoing IVF with euploid embryos [77]. The analysis revealed the most abundant genera to be *Lactobacillus* and *Flavobacterium*, consistent with previous findings of other genital tract bacteria. However, no statistically significant correlation was found between these patients' endometrial microbiome profile and reproductive outcomes [77]. Like *Brevundimonas*, *Flavobacterium* has been shown to produce carotene and exhibit catalytic activities. This suggests that *Flavobacterium* may have the ability to mitigate seminal oxidative stress by reducing the damage caused to cell membranes through lipid peroxidation. In this scenario, DNA damage becomes a concern as DNA is more susceptible to hazards. Additionally, disruption to the cell membrane can have an impact on sperm viability [78].

The similarities between the endometrial and vaginal microbiota support the hypothesis that the uterus is colonized by bacteria ascending from the vagina; however, the differences observed in some cases suggest that this process is not identical in every woman. Comparative studies of the endometrial microbiome in healthy and infertile women have highlighted significant differences in bacterial composition and diversity between the two groups [45, 70, 75]. These findings suggest that disruptions in the normal endometrial flora may contribute to infertility, potentially through mechanisms such as increased inflammation, compromised endometrial receptivity, and impaired embryo implantation [70]. Therefore, further research is needed to elucidate the exact role of endometrial dysbiosis in female infertility and to determine the prevalence of infertility attributable to this condition [45]. This knowledge may aid in the development of personalized treatment strategies to address the specific microbial imbalances contributing to a patient's infertility [45, 79].

### 3.6 The Intestinal Microbiome and Infertility

Infertility is a complex condition that affects millions of couples worldwide. Recent studies have suggested that the gut microbiome may play a role in infertility [80]. The gut microbiome is a complex ecosystem of microorganisms that reside in the gastrointestinal tract and play a crucial role in maintaining human health [81]. Alterations in the gut microbiome have been associated with various diseases, including inflammatory bowel disease, obesity, and diabetes. However, the relationship between the gut microbiome and infertility is not yet fully understood [80].

The study discovered that women with recurrent implantation failure and infertility had intestinal dysbiosis and a higher incidence of ubiquitin in their vaginal microbiome. In contrast, the control group exhibited the reverse pattern [82]. Furthermore, a separate study looked at worldwide changes in the gut flora of reproductive‐aged women who had constipation. Significant variations in the intestinal microbiota between constipated and healthy women were discovered, such as higher bacterial abundance and lower Proteobacteria prevalence. Both studies contribute to our understanding of gut and vaginal microbiota in women of reproductive age, but neither explicitly addresses the link between gut microbiome and infertility, underscoring the need for more study in this area [83].

PCOS is a complex disorder that affects the endocrine, neuroendocrine, and metabolic systems, leading to difficulties with pregnancy and infertility [84]. To better understand this condition, researchers have analyzed the gut microbiome of individuals with and without PCOS. One such study conducted by Lowell et al. compared stool samples from 102 patients with PCOS and 201 healthy individuals matched for age and body mass index (BMI). The researchers identified four bacterial genera that were significantly different between the two groups. Specifically, the abundance of two genera from the *Clostridiales* order (*Ruminococcaceae* UCG‐002 and *Clostridiales* family XIII AD3011) was correlated with markers of PCOS, such as cystic ovaries and high testosterone levels. Additionally, patients with PCOS and prediabetes had lower alpha diversity (as measured by Shannon's index) and higher abundance of the genera *Dorea* and *Bacteroides* ( *Ruminococcus torques* group and *Lachnospiraceae* UCG‐004) compared to PCOS patients with normal glucose tolerance [85]. *Ruminococcaceae* in the gut have been associated with the production of short‐chain fatty acids in overweight and obese individuals with PCOS. Additionally, in individuals with type 2 diabetes, they may contribute to the production of inflammatory cytokines [86]. In summary, although there are indications that the gut microbiome, endometrial microbiome, and female genital tract microbiome may contribute to infertility (Figure 1), the specific bacterial species that require augmentation or reduction in the microbiome of infertile women remain unclear. Additional investigations are necessary to ascertain a more specific association between the microbiome and infertility. Additionally, further research is warranted to elucidate the role of the microbiome in endometrial obstetrical disorders and to delineate and comprehend the function of the female genital tract microbiome in fertility [80] (Table 1).

**TABLE 1:** The role of the microbiome in endometrial obstetrical disorders.

References	Subjects	Sample	Microbiota species	Results	Conclusion
[84]	47 PCOS patients and 50 healthy reproductive‐aged controls	Vaginal swabs	*Lactobacillus* spp. *Gardnerella vaginalis* , *Prevotella*, *Mycoplasma hominis*	Several non‐*Lactobacillus* taxa, including *Gardnerella vaginalis* , *Prevotella*, and *Mycoplasma hominis* , were found to be more abundant in the LGT microbiota of PCOS patients	There is a difference between the microorganisms in the LGT of patients with PCOS and healthy reproductive‐aged women
[87]	Women with newly diagnosed PCOS (*n* = 39) and healthy controls (*n* = 40)	Vaginal swabs	*Lactobacillus crispatus* , *Mycoplasma genus*	*The Mycoplasma genus could serve as a potential biomarker for screening PCOS*	In the vaginal microbiome, the *Mycoplasma* genus is associated with PCOS
[88]	47 PCOS patients and 50 control reproductive‐aged women	Vaginal and cervical swabs, cervical epithelial cells samples	*Lactobacillus*	The gene expression profiles of the cells significantly differed between the PDB group (PCOS patients with a relative abundance of *Lactobacillus* < 50%, *n* = 4) and the PNB group (PCOS patients with a relative abundance of *Lactobacillus* ≥ 50%, *n* = 5)	The potential relationship between the LGT microbiota and reproductive system function, as well as IVF‐FET outcomes, in PCOS patients was elucidated
[89]	PCOS group, *n* = 713, control group, *n* = 733	Vaginal swabs	*Lactobacillus*, *Gardnerella*, *Ureaplasma*	The proportion of *Lactobacillus* in the PCOS group decreased, while the proportions of *Gardnerella* and *Ureaplasma* increased	The PCOS group exhibited a higher diversity of vaginal microbiome and demonstrated an increased level of heterogeneity
[90]	24 PCOS patients and 20 healthy controls	Saliva samples	*Phylum Actinobacteria*	Patients with PCOS exhibited a decreased salivary relative abundance of *Actinobacteria*	Association of the saliva microbiome of PCOS patients with PCOS‐related parameters
[91]	42 PCOS patients and 24 healthy controls	Vaginal swabs	*Lactobacillus*	There were statistically significant differences in the vaginal microbiome between PCOS patients and healthy women, regardless of the abundance of *Lactobacillus*	*Actinomyces* could potentially serve as a biomarker to identify PCOS
[92]	89 female patients with PCOS	Vaginal swabs	*Lactobacillus crispatus* , *Lactobacillus iners*	The abundance of *Lactobacillus crispatus* was higher (*p* = 0.010), while that of *Lactobacillus iners* was lower (*p* = 0.036), among PCOS patients with elevated testosterone levels	The vaginal bacterial species among PCOS patients with varying clinical manifestations, particularly differences in testosterone levels, are distinct
[93]	PCOS and obese, PCOS and non‐obese, non‐PCOS and obese, and non‐PCOS and non‐obese	Swab specimens	*Streptococcus pyogenes* , *Leptospira santarosai* , *Citrobacter amalonaticus* , *Listeria ivanovii* , and *Clostridium perfringens*	In addition to *Lactobacillus* bacteria, *Lactobacillus* phage and *pseudomonas* phage/virus were identified as indicators of a healthy vaginal microbiome	PCOS and obesity exhibit distinct enrichments of bacteria and viruses/phages, and both conditions are associated with microbial dysbiosis
[94]	18,340 individuals	The GWAS data for PCOS comprised 113,238 samples	*Actinomyces*, *Streptococcus* and *Ruminococcaceae*, *Candidatus Soleaferrea Ruminococcaceae*	Changes in gut microbiota at various taxonomic levels were determined in PCOS patients	There is a causal relationship between the gut microbiome and PCOS, which may contribute to providing novel insights for the development of new preventive and therapeutic strategies for PCOS
[95]	260 participants	Vaginal samples	*Lactobacillus*	The efficacy of transvaginal *Lactobacillus* supplementation in restoring the LGT microbiome and improving perinatal outcomes in PCOS patients after IVF‐FET was examined	This holds promise for increasing the rates of clinical pregnancy and live birth in PCOS patients after IVF‐FET
[96]	703 women with endometrial polypoid lesions and 703 women in the control group	Vaginal samples	*Lactobacillus crispatus* , *Leptotrichia* spp. and *Cutibacterium* spp., *Fannyhessea* spp., *Acinetobacter* spp. and *Achromobacter* spp.	The control group exhibited a higher relative abundance of *Lactobacillus crispatus* than the polypoid lesions group	The associations between vaginal microbiota and endometrial polypoid lesions were elucidated, highlighting the potential relationship between a well‐balanced vaginal microbial ecosystem and a healthy intrauterine environment
[97]	23 couples with idiopathic infertility	Vaginal swabs and seminal fluids	*Lactobacillus crispatus* , *Lactobacillus iners* , and *Lactobacillus gasseri*	Idiopathic infertile women exhibited a different average composition of the vaginal microbiome compared to control sequences, while no significant differences were observed in the seminal counterpart	Microbiome characterization could be useful, along with standard clinical and laboratory assessments, in the pre‐IUI evaluation of infertile couples
[98]	Recurrent Spontaneous Abortion group (*n* = 25) and the control group (*n* = 25)	Endometrial tissue samples and uterine lavage fluid	Microbiota of the lower and upper female reproductive tracts	The microbiota of the lower and upper female reproductive tracts from patients with RSA showed no significant differences in alpha diversity compared to controls	Alterations in the microbiota in the uterine cavity could be associated with changes in cytokine levels, which may be a risk factor for RSA pathogenesis
[99]	18,340 individuals	Two‐sample MR analysis	*Lachnospiraceae UCG001* and *Ruminococcus 2*, *Butyricicoccus*, and *Prevotella*	The relationship between gut microbiota and inflammation in specific pelvic organs such as salpingitis, oophoritis, vulvar, or vaginal inflammation was identified	Investigating the causal relationship between gut microbiota and female reproductive health
[100]	Infertile women with (*n* = 20) or without chronic endometritis (*n* = 103)	Vaginal secretions	*Bifidobacterium* and *lactic acid‐producing bacteria*	The detection rate of *Streptococcus* and *Enterococcus*, as well as the bacterial abundance of *Atopobium* and *Bifidobacterium*, in the vaginal secretions microbiota, was significantly lower in the chronic endometritis group than in the non‐chronic endometritis group	The vaginal secretions microbiota in infertile women with CE is characterized by a reduction in *Bifidobacterium* and lactic acid‐producing bacteria, apart from *Lactobacillus*

Abbreviations: CE, chronic endometritis; GWAS, genome‐wide association study; IUI, intrauterine insemination; IVF‐FET, in vitro fertilization frozen embryo transfers; LGT, lower genital tract; MR, miscarriage rate; PCOS, polycystic ovary syndrome; RSA, recurrent spontaneous abortion; VS, vaginal secretions.

### 3.7 Oral Microbiota and Infertility

The oral microbiota plays an important role in maintaining healthy vaginal and reproductive tract environments in women [53]. Dysbiosis of the oral microbiota has been linked to diverse gynecological disorders, including infertility [53, 101]. As an illustration, a study conducted in 2016 investigated the oral microbiota of healthy women with normal fertility and women with unexplained infertility [102]. The infertile women had significantly higher levels of Proteobacteria, particularly Enterobacteriaceae, and lower levels of *Fusobacteria* and *Leptotrichia* in their oral samples [102, 103]. Normally nonpathogenic oral anaerobic bacteria, *Fusobacterium sp*. has been suggested to move hematogenously to the placenta and alter the permeability of the vascular endothelium, which may facilitate the colonization of other potentially pathogenic organisms like *Pseudomonas* spp. [104]. These bacterial alterations were correlated with elevated serum levels of cortisol, a stress hormone, in infertile women [105]. Restoring eubiosis of the oral microbiome through probiotic supplementation led to improved fertility parameters in some infertile women [106]. Some key bacteria that may influence fertility potential include *Porphyromonas gingivalis* ( *P*. *gingivalis* ), *Treponema denticola* ( *T*. *denticola* ), *Tannerella forsythia* ( *T*. *forsythia* ), *Streptococcus sanguinis* ( *S*. *sanguinis* ), and *Streptococcus mitis* ( *S*. *mitis* ) [107]. Overgrowth of *P*. *gingivalis* , *T*. *denticola*, and *T*. *forsythia* , commonly known as the “red complex,” has been linked to elevated inflammation and menstrual cycle irregularities, increasing the risk of infertility. In contrast, *S*. *sanguinis* and *S*. *mitis* promote a healthy oral environment and vaginal microbial balance [108]. Fertility may be improved by taking steps to balance the microbiota and improve dental health [109]. This comprises the following:

1. Using a probiotic oral supplement or fluoride rinse [109].
2. Improving oral hygiene with regular brushing and flossing [110].
3. Reducing antibiotic use and sugar consumption.
4. Controlling periodontal disease with scaling, root planning, or, if necessary, surgery [111].
5. Using relaxation techniques to reduce cortisol levels [109].

Addressing oral dysbiosis through integrated approaches including both oral health and gynecologic care may help promote fertility and reproductive wellness in women [112].

### 3.8 Endocervical Microbiome and Infertility

The endocervical microbiome, which refers to the microorganisms within the cervix, has been shown to play a role in female reproductive health [113]. Recent studies have shown that infertile women have a different endocervical microbiome composition compared to healthy individuals, with a higher prevalence of pathogenic bacteria such as *C*. *trachomatis* [27, 114]. Likewise, an investigation revealed that women with endometriosis‐related infertility displayed a disparate endocervical microbiota composition compared to those without endometriosis, indicating a potential relationship between endocervical dysbiosis and infertility [113, 114]. An additional investigation uncovered that the endocervical microbiota composition fluctuated over the menstrual cycle, with a marked decline in the prevalence of *Lactobacillus* spp. during menstruation. These findings imply that the endocervical microbiota may be especially susceptible to alterations during menstruation, which could potentially impact female reproductive health [115]. It has also been shown that women with bacterial vaginosis, characterized by an overgrowth of pathogenic bacteria, had a higher prevalence of endocervical infections and adverse pregnancy outcomes [116]. Common bacteria in the endocervical microbiome include *Lactobacillus*, *Gardnerella*, and *Streptococcus* [27, 117]. The relative abundance of *Lactobacillus* in the endocervix of infertile women is significantly lower than that of healthy individuals, while the relative abundance of *Gardnerella* and *Streptococcus* is higher [118]. Researchers found in one study that women with recurrent miscarriages had a significantly different endocervical microbiome composition than women with a history of successful pregnancies, suggesting a possible link between endocervical dysbiosis and pregnancy outcomes [114, 117]. The endocervical microbiota composition was linked to PCOS, a frequent cause of infertility in women [119]. In particular, the study discovered that the endocervical microbiome composition of women with PCOS was different from that of healthy controls, with a reduced abundance of *Lactobacillus* and a higher abundance of *Streptococcus*. These results imply a potential role for the endocervical microbiome in the pathophysiology of PCOS and subsequent infertility in affected women (Table 2) [119].

**TABLE 2:** The role of the endocervical microbiome in the pathophysiology of PCOS and infertility.

Author/(year)/(reference)	Country	Type of study	Demographics of clinical trial participants	Sample type	Detection method	Outcome
Moreno et al. (2022) [74]	13 reproductive clinics in Asia, America, and Europe	Prospective observational study	Infertile women undergoing ART treatment: 342	EB and EF	NGS	The profile of endometrial microbiota in women with successful pregnancy has a direct relationship with an increase in *Lactobacillus* dominance. Conversely, the dysbiotic profile of endometrial microbiota associated with infertility includes *Staphylococcus*, *Klebsiella*, *Haemophilus*, *Gardnerella*, *Atopobium*, *Bifidobacterium*, *Chryseobacterium*, and *Streptococcus*
Diaz‐Martinez et al. (2021) [120]	Spain	Pilot prospective cohort	Women undergoing IVF: 48	Vaginal samples, Tao Brush IUMC Endometrial Sampler	NGS	There was a statistically significant difference in terms of alpha and beta diversity in the endometrial microbiota between women with and without RIF
Azpiroz et al. (2021) [29]	USA	Observational, exploratory, preliminary	IVF failure in infertile patients: 287 Controls: 20	Vaginal and rectal swap	NGS	The fertile group exhibited an increase in the ratio of *Lactobacillus brevis* / *Lactobacillus iners* and Firmicutes/Bacteroidetes, while the infertile group had lower bacterial richness
Carosso et al. (2020) [121]	Italy	Pilot	Infertile women: 15	Catheter tip and vaginal swab	NGS	Control of VM through progesterone supplementation and controlled ovarian stimulation can impact endometrial and placental receptivity. This may involve a reduction in the *Lactobacillus* ratio and an increase in *Prevotella*, *Escherichia coli* , *Shigella* spp., and *Atopobium* in the samples taken
Komiya et al. (2020) [122]	Japan	Observational	Infertile women: 18 Controls: 18	Feace sample	Illumina MiSeq sequencing of V3–V4 regions of the 16S rRNA gene	A decrease in the abundance of *Paraprevotella* and *Blautia*, coupled with an increase in the abundance of *Bifidobacterium*, can serve as a predictor of pregnancy
Kairi Koort (2023) [123]	Estonia	Observational	ART couples: 97 Healthy couples: 12	Vaginal and semen samples	16S rDNA sequencing	The success rate of ART was lower in women with the dominant microbiome of *L*. *iners* and *L*. *gasseri* in the presence of bacterial vaginosis compared to women with the dominant microbiome of lactic acid bacteria and *L*. *crispatus*
Cheong et al. (2019) [117]	Malaysia	Cohort	Infertile women: 34	Endocervical swabs	16SrRNA metagenomic sequencing	Evaluation of endocervical microbiome changes revealed *Chlamydia trachomatis* infection in 88% of infertile women and 28% of the fertile group
Kyono et al. (2019) [124]	Japan	Case–control and prevalence	IVF patients: 79 Non‐IVF infertile: 23 Controls: 7	EF	16s rRNA V4 Illumina MiSeq Greengenes database v13_8	A lower percentage of LD was observed. Endometrial morphology remains stable during and between menstrual cycles. The average percentage of LD and non‐NLD endometrial microbiota in pregnant patients is 96.5% (± 34%) and 39%, respectively. Other dominant bacterial genera in NLD patients included *Gardnerella*, *Streptococcus*, *Atopobium*, *Bifidobacterium*, *Sneathia*, *Prevotella*, and *Staphylococcus*
Kitaya et al. (2019) [125]	Japan	Case–control and transversal Descriptive	RIF patients: 28 Patients no RIF: 18	Vaginal swab and EF	PCR analysis	The vaginal microbiota dominated by LD in the RIF group (67.9%) was higher than that of the control group (44.4%). The detection rate of Gardnerella in the EF microbiota was higher in the RIF group (39.3%) compared to the control group (27.7%). Additionally, *Burkholderia* was found only in the EF microbiota of the RIF group (25%) and not in the control group
Liu et al. (2019) [126]	China	Observational	Infertile women: 130	EF and EB	RNA sequencing	18 types of bacteria, including *Dialister*, *Bifidobacterium*, *Prevotella*, *Gardnerella*, and *Anaerococcus*, were found to be more abundant in the endometrial microbiota of infertile women with chronic endometritis. Among these, *Anaerococcus* and *Gardnerella* exhibited a negative correlation in relative abundance with *Lactobacillus*
Bernabeu et al. (2019) [127]	Spain	Cohort	Patients treated with ART: 31	Vaginal samples	16S rRNA analysis	There were no significant differences between the pregnant and non‐pregnant groups in terms of alpha diversity, beta diversity, *lactobacillus* dominance flora, or bacterial dominance (especially in *Gardnerella* spp.). However, patients who achieved pregnancy had lower values of the Chao1 index
Koedooder et al. (2019) [128]	Netherlands	Cohort	Women undergoing IVF‐ICSI or IVF treatment: 192	Vaginal swab	(IS‐pro) technique	The presence of LD flora was associated with higher pregnancy rates, while the presence of *L*. *crispatus* , *L*. *jensenii* , *Proteobacteria* spp., and *Gardnerella vaginalis* was associated with lower pregnancy rates
Hashimoto et al. (2019) [129]	Japan	Cohort	Patients with blastocyst SET: 99	EF	16SrRNA metagenomic sequencing	There were no significant differences found between groups E and D in terms of IR, PR, or MR. The most common genera of bacteria found in the dysbiotic endometrial microbiome were *Atopobium*, *Gardnerella*, and *Streptococcus*. The same microbiome composition was observed in both patients who conceived and those who did not
Amato et al. (2020) [97]	Italy	Cohort and case–control	Patients: 23	Vaginal swab	16SrRNA metagenomic sequencing	There is no connection between VM and idiopathic infertility. However, a lower diversity of microbiota, with a higher abundance of *L*. *crispatus* and a lower amount of *Bifidobacterium* spp., was found to be associated with a higher chance of pregnancy after IUI
Wee et al. (2018) [76]	Australia	Case–control	Infertile: 15 Fertile: 16	Endometrial, vaginal cervical samples	RNA sequencing PCR analysis	It has been shown that infertile women have a higher prevalence of *Ureaplasma* bacteria in their vaginal microbiome and *Gardnerella* bacteria in their cervical microbiome
Graspeuntner et al. (2017) [130]	Germany	Case–control	Fertile patients: 89 Non‐infectious infertility women: 26 Infectious infertility women: 21 Female sex workers: 54	Cervical swabs	Culture/PCR 16S amplicon sequencing	Infertile patients with an infectious cause had a lower abundance of *Lactobacillus* bacteria. Instead, there was a higher presence of *Gardnerella*/ *L*. *gasseri* bacteria in their microbiome, while *L*. *crispatus* was more commonly found in fertile patients
Babu et al. (2017) [131]	India	Comparative	Infertile women: 116	Vaginal swabs	Cultivation of bacteria	The dominant flora in asymptomatic vaginosis patients consisted of *Candida* spp. and *Enterococcus*, with *Escherichia coli* (14%) and a very low percentage of *Lactobacillus* present in 28% of cases
Tao et al. (2017) [132]	United States	Cross‐sectional	Infertile women: 70	The distal end of the embryo transfer catheter	RNA sequencing/PCR analysis	*Lactobacillus* spp. were detected in all samples. Additionally, *Corynebacterium*, *Bifidobacterium*, *Staphylococcus*, and *Streptococcus* spp. were also detected in some patients
Haahr et al. (2018) [23]	Denmark	Cohort	The number entered in the analysis: 75	Vaginal swab	16s rRNA/qPCR	The composition of the VM was not associated with differences in pregnancy rates. However, higher diversity in the EM was linked to lower clinical pregnancy and live birth rates
Franasiak et al. (2016) [77]	United States	Cohort	Patients: 33	Transfer catheter	16S ribosomal subunit hypervariable region analysis with NGS	*Lactobacillus* was identified as the most common species in both groups of pregnant women and non‐pregnant women
Moreno et al. (2016) [133]	Spain	Descriptive and cohort	Infertile patients: 35	Vaginal aspirate and EF	RNA sequencing	*Lactobacillus* was the most prevalent genus (72%), followed by *Gardnerella* (12.6%), *Bifidobacterium* (3.7%), *Streptococcus* (3.2%), and *Prevotella* (0.866%). NLD in the endometrial microbiota was associated with significant reductions in implantation, pregnancy, ongoing pregnancy, and live birth rates
Campisciano et al. (2017) [134]	Italy	Case–control	Infertile women attending the ART: 23	Vaginal sample	V3‐16S rDNA sequencing	The abundance of *L*. *gasseri* and the absence of *L*. *iners* and *L*. *crispatus* in the VM, along with the presence of *Veillonella* spp., *Staphylococcus* spp., *Gardnerella vaginalis* , *Atopobium vaginae* , *Prevotella bivia* , and *Ureaplasma parvum* , were associated with idiopathic infertility

Abbreviations: ART, assisted reproductive technology; CPR, clinical pregnancy rate; CST, community state type; D, dysbiotic; E, eubiotic; EB, endometrial biopsy; EF, endometrial fluid; EM, endometrial microbiome; IR, implantation rate; IS‐pro, interspace profiling; IUI, intrauterine insemination; IVF, in vitro fertilization; LBR, live birth rate; LD, *Lactobacillus* dominant; MR, miscarriage rate; NGS, next‐generation sequencing; NLD, non‐*Lactobacillus* dominated; PR, pregnancy rate; RIF, recurrent implantation failure; SET, single embryo transfer; VM, vaginal microbiota; VS, vaginal secretions.

## 4 Microbiota in the Male Reproductive System

The group of bacteria that reside both within and outside of the human body is known as the human microbiome [135]. This complex collection of microorganisms is essential to human health as it has co‐evolved with us and is involved in many physiological functions. Everybody has a different microbiota, with different species inhabiting different body parts [136].

Previously thought to be mostly sterile, the male reproductive system is now understood to be a complex mosaic of microbial communities. Different microbial communities are present in the male genital canal, which includes areas like the urethra and the coronal sulcus. In the male reproductive system, *Corynebacterium*, *Streptococcus*, and *Staphylococcus* are the predominant bacterial genera [137]. Individual differences may be seen in the microbial makeup of this tract, which can be impacted by things like sanitary habits, sexual behavior, and the existence of sexually transmitted infections (STIs) [138].

Further investigation reveals that the testes, which were previously thought to be completely germ‐free, actually have a modest quantity of microbiota. Particular bacterial communities are also seen in the epididymis, a duct where sperm develop; the importance of these communities is currently being studied. The microbiota of the prostate and seminal vesicles, two glands essential for the formation of seminal fluid, might affect the fluid's composition and overall health. The urethra, which connects to the outside world, has a varied microbiota that affects its well‐being. In addition to sperm, semen also carries microbial communities that can impact the fertility and health of sperm. Last but not least, the microbiota of the penile skin, which includes the glans and shaft, is altered by things like circumcision [136]. Optimal reproductive health requires a varied and well‐balanced microbiota in the male genital system [137]. The male reproductive system may be harmed and chronic prostatitis may be caused by bacteria such as *E*. *coli* and *Ureaplasma urealyticum* ( *U*. *urealyticum* ), which could cause inflammation [139]. The microbial makeup of the male urinary system can also affect a person's vulnerability to STIs and the likelihood of the infection spreading to a partner [140].

### 4.1 Microbial Dysbiosis and Male Infertility

#### 4.1.1 Definition and Characterization of Microbial Dysbiosis

Any change in the composition and activity of microbial communities within the human microbiome is referred to as microbial dysbiosis. It is characterized by a departure from the normal or ideal microbial composition, which causes modifications to the metabolic processes and component distributions within the microbiota [141]. A contributing cause to male infertility is thought to be dysbiosis in the microbiota of the male reproductive system. Precision medicine depends on the identification of specific microbial imbalances, which allows for customized therapies to address the underlying reasons of male infertility [142]. Male reproductive tract dysbiosis is complicated and varies between illnesses and people. It frequently results in a rise in facultative anaerobic species and a decrease in microbial diversity [141]. The quality and functioning of sperm can be negatively impacted by such dysbiosis, which can have a negative influence on the male reproductive system [136]. The development of male infertility is substantially influenced by oxidative stress. The risk of infertility is increased by elevated oxidative stress levels or DNA‐damaged sperm [143]. Infertility, urethritis, and prostatitis have all been linked to dysbiosis of the male genital tract microbiota. Certain bacterial species, such as *U*. *urealyticum* and *E*. *coli* , have been connected to inflammation in the male reproductive system and chronic prostatitis [31].

#### 4.1.2 Evidence Linking Microbial Dysbiosis to Male Infertility

**FIGURE 2:**
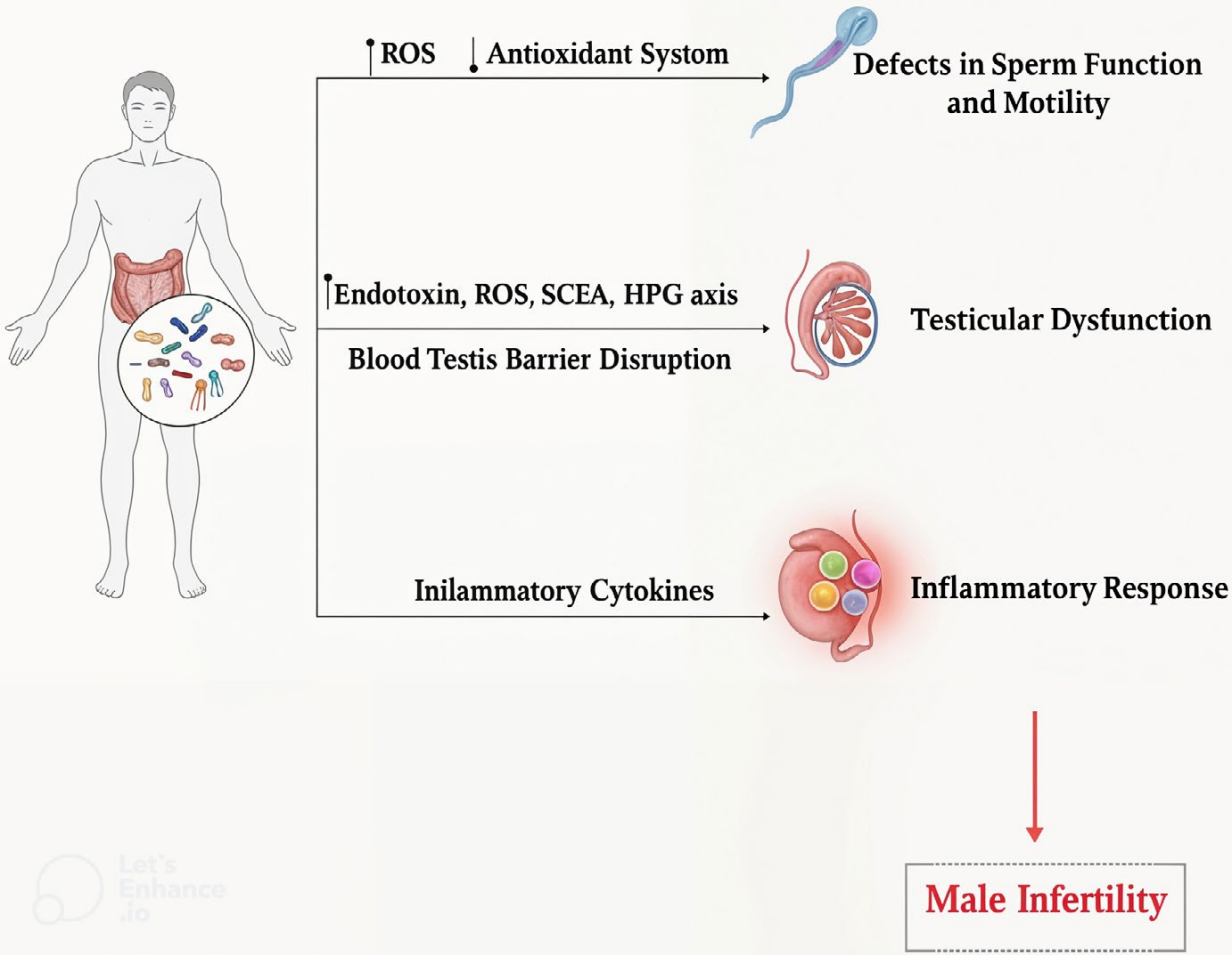
Effect of microbial dysbiosis on male infertility.

Through various processes such as inflammation, oxidative stress, decreased sperm function and motility, and disrupted testis function, microbial dysbiosis can have a deleterious impact on male fertility (Figure 2).

Maintaining redox equilibrium is crucial for sperm functioning in several critical areas. However, an imbalance in the production and removal of reactive oxygen species (ROS) due to oxidative damage can detrimentally affect sperm quality [144]. Semen samples from infertile patients often exhibit significantly elevated ROS levels, found in 25%–40% of cases [145]. Depending on the amounts and characteristics of reactive molecules, the duration of exposure, the effectiveness of antioxidants, ambient temperature and oxygen tension, and other factors, the degree of oxidative damage to spermatozoa can vary greatly among infertile men. Prolonged exposure to high concentrations of ROS can damage various essential cellular macromolecules, such as proteins, lipids, and nucleic acids, ultimately impairing several cellular processes [146].

Dysregulated gut microbiota stimulates dendritic cells and macrophages in the testis, leading to increased release of pro‐inflammatory substances. Men experiencing infertility often exhibit chronic inflammation in the male reproductive system, exacerbating fertility issues, as evidenced by epidemiological studies [147]. Experimental models of autoimmune orchitis further elucidate this association, demonstrating significant macrophage infiltration and the release of numerous inflammatory markers. These findings underscore the complex interaction between inflammation and male reproductive health [148].

The gut microbiota influences male reproductive activity, subsequently impacting the host's physiology and fitness through the microbiota–gut–testis axis. Exploring novel treatments for testicular dysfunction by modulating the gut microbiota is particularly intriguing. The gut microbiota can be altered by androgens or other communication molecules secreted by the testis. Conversely, endotoxins, induced ROS, metabolites (such as SCFA), regulation of gut metabolism and nutrient intake, or the hypothalamic–pituitary–gonadal (HPG) axis are mechanisms through which the gut microbiota influences testicular function. Probiotics, along with other microbial regulators, medications, toxins, nutrition, and lifestyle choices, can all influence the gut microbiota [149].

#### 4.1.3 The Impact of Gut Microbial Dysbiosis on Male Infertility

Sperm concentration and motility were clearly reduced in mice fed a high‐fat diet, according to Ding et al. [150]. Furthermore, the gut microbiota of these animals exhibited an increase in Firmicutes and Proteobacteria and a decrease in Bacteroidetes and Verrucomicrobia. After transplanting the fecal microbiota from mice treated with alginate oligosaccharide to mice treated with busulfan, Zhang et al. [151] observed a noteworthy increase in sperm concentration and motility. Specifically, Bacteroidales and Bifidobacteriales populations of “beneficial” bacteria increased in response to this modification. Alginate oligosaccharides have been shown by Zhao et al. [152] to have the ability to mitigate the inhibition of spermatogenesis in mice brought on by busulfan. An increase in *Lactobacillaceae* and Bacteroidales, two types of good bacteria, and a reduction in *Desulfovibrionaceae*, a kind of harmful bacteria, were associated with this impact.

Artificial diets have been shown to cause microbiota dysbiosis in mice and to negatively impact their reproductive systems. Spermatogenesis defects have also been linked to elevated endotoxins, dysregulated testicular gene expression, and localized epididymal inflammation. Sex hormones have been shown to play a role in the communication between microorganisms and hosts and to influence host reproduction [153]. Dysbiosis of the gut microbiota is linked to the regulation of host hormone levels through close communication between the gut and testicular tissues, so preventing reproduction in wild animals [154].

Staphylococcal species, which live in harmony with humans, have the ability to directly damage reproductive tissues or to do so through hematogenous pathways. They may also orchestrate the inflammatory response in the reproductive system by way of an innate immune route that is activated by TLR2 [155]. *S*. *aureus* is the species of Staphylococcus that is most closely associated with male infertility. Because *S*. *aureus* increases aberrant morphology and decreases sperm concentration, it may be a significant negative factor contributing to the decline of male reproductive function [156]. Furthermore, it has been found that aberrant sperm are linked to elevated *S*. *aureus* concentrations in seminal vesicles. In the treatment of male infertility, colonization of *S*. *aureus* in the male reproductive system should not be disregarded as it can also immobilize and agglutinate spermatozoa [157].

One of the most prevalent sexually transmitted infections of the male reproductive system is *C*. *trachomatis* [158]. The detection rate of *C*. *trachomatis* in infertile men was found to be several times higher than in healthy fertile men [159]. Numerous studies support the detrimental effects of *C*. *trachomatis* infection on sperm count, motility, normal morphology, production of reactive oxygen species, total antioxidant capacity, and the ability of sperm from infertile men to penetrate eggs. Additionally, *C*. *trachomatis* infection may increase the incidence of male infertility by causing the synthesis of anti‐sperm antibodies [157]. One of the most well‐known sexually transmitted infections is the HPV, which can cause cancers linked to HPV in both men and women. Harmful effects of HPV infection on male infertility have also been reported, including decreases in sperm count, motility, volume, and normal sperm morphology, which suggests that HPV infection is a risk factor for male infertility. However, other viruses, including adeno‐associated virus, cytomegalovirus, and herpesviruses, have also been linked to male infertility; however, the evidence currently available does not clearly link these factors to male fertility [160].

A study conducted by Tian et al. [161] revealed that *Candida albicans* ( *C*. *albicans* ) inhibits human sperm motility and disrupts the ultrastructure of human spermatozoa in vitro, potentially linking it to male infertility. Similarly, a study by Burrello et al. (2004) demonstrated that the presence of *C*. *albicans* increased sperm DNA debris and hindered oocyte fertilization. The relationship between fungal communities, particularly *C*. *albicans* , and male infertility is an area of research that is still being explored and understood [162].

#### 4.1.4 Microbial Dysbiosis in the Reproductive System and Its Association With Male Infertility

Male fertility can be impacted by a wide range of variables, such as microbial dysbiosis, immunological interactions, metabolic processes, and STIs [137]. Male microbiome‐produced metabolites may have an immediate or long‐term impact on the reproductive system, which may impair fertility. Aside from sperm quality, other parameters might also be affected by such microbial imbalances [163]. Couple fertility may be impacted by the “seminovaginal microbiota,” which is transferred between partners during intercourse. Both couples' microbiota should be taken into account in thorough fertility evaluations [164]. The *Prevotella* genus has been associated with poor‐quality semen in analyses of human semen samples, indicating that some *Prevotella* species may be to blame for impaired spermatogenesis and male infertility. Given that *Prevotella* in semen comes straight from the testis, the finding that its quantity in semen was negatively linked with semen concentration [25] is particularly noteworthy. Moreover, there is a clear correlation between *Prevotella* abundance and BMI, indicating that it might be a factor in obesity‐related infertility [165]. Furthermore, *Pseudomonas* spp. are expressed in greater abundance in the semen of infertile patients. *Pseudomonas* abundance has a direct correlation with the total number of motile sperm [37]. Additionally, it was recently shown that males with aberrant sperm concentrations have greater *Pseudomona*s and *Pseudomonas fluorescens* abundances in their semen [166].

#### 4.1.5 Dysbiosis in Specific Regions of the Male Reproductive System

##### 4.1.5.1 Rete Testis, Efferent Ducts, and Epididymis

There is a shortage of research on the testicular microbiota. Changes in the testicular microbiota have been linked to male infertility, according to preliminary research by Alfano et al. Infertile men have lower levels of Proteobacteria and Bacteroidetes in their testicles. The microsurgical testicular sperm extraction tests produced no sperm, but the *Firmicutes* and *Clostridium* abundances changed, *Peptoniphilus asaccharolyticus* was completely absent, and Actinobacteria was elevated [167].

##### 4.1.5.2 Deferent Duct, Seminal Vesicles, and Prostate

Following a vasectomy, Lundy et al. [168] found that the amount of *Collinsella* (phylum Actinobacteria) and *Staphylococcus* (phylum Firmicutes) in semen was reduced. This finding suggests a possible connection between male infertility and the microbiota of the testicles.

##### 4.1.5.3 Urethra and Coronal Sulcus

There are several microbial populations in the male genital canal, which includes areas like the urethra and the coronal sulcus. In the male reproductive system, *Corynebacterium*, *Streptococcus*, and *Staphylococcus* are the predominant bacterial genera [169].

##### 4.1.5.4 Semen

Research on male infertility has primarily focused on semen samples, consistently revealing differences in the microbiota composition of infertile male semen. Notably, there is an increased abundance of *Prevotella* and *Staphylococcus*, and a decreased abundance of *Lactobacillus* and *Pseudomonas*. Studies by Baud et al. [170] and Farahani et al. [171] have highlighted a negative correlation between *Prevotella* prevalence and sperm motility, while a reduced *Lactobacillus* abundance was directly linked to abnormal sperm morphology. Comparisons of rectal samples from infertile men with those from fertile individuals have shown variations in the abundance of *Anaerococcus* and an elevated presence of *Lachnospiracea*. *Anaerococcus*, conversely, was found in higher concentrations in urine samples from infertile males. Additionally, *Collinsella* was less prevalent in semen samples from infertile men, while *Aerococcus* was more prevalent. Further investigations revealed a statistically significant inverse correlation between leukocytospermia and semen viscosity and the quantity of *Aerococcus*. Furthermore, a statistically significant inverse relationship between the concentration of semen and *Prevotella* abundance was found. On the other hand, *Pseudomonas* abundance and sperm count were found to have a statistically significant positive connection, but with inverse proportionality to semen pH. Further substantial longitudinal research across different institutions is necessary to validate the results of these investigations [168].

### 4.2 Diagnostic Approaches for Assessing Microbial Dysbiosis in Infertility

**FIGURE 3:**
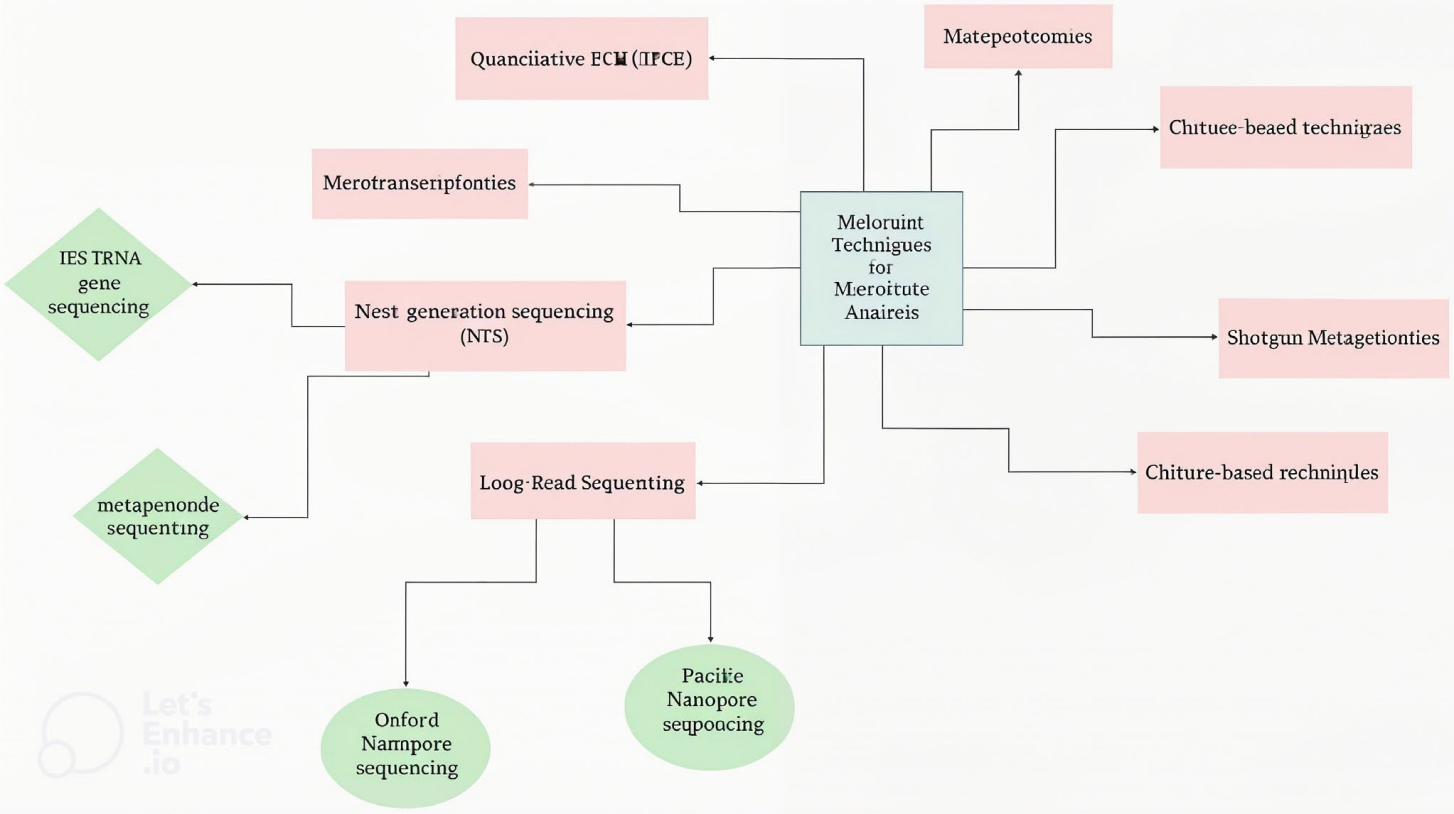
Molecular techniques to analyze the composition and diversity of the microbiota.

Current methods generally focus on the investigation of microbiota composition and diversity utilizing techniques including culture‐based techniques, PCR, quantitative PCR (qPCR), and next‐generation sequencing (NGS) to determine microbial dysbiosis in infertility. In culture‐based approaches, bacteria are isolated and cultivated from clinical samples, and then they are identified using DNA sequencing or biochemical testing. Nonetheless, these techniques frequently exhibit a bias toward culturable bacteria and cannot fully capture the diversity of microbial populations (Figure 3).

## 5 Microbial Biomarkers and Infertility

### 5.1 Age

The health of the reproductive tract environment and the regular functioning of the reproductive endocrine system are both essential for women of reproductive age to achieve a successful pregnancy. Reproductive health, fertility maintenance, and gonadal development all rely on reproductive endocrine function. Simultaneously, maintaining pregnancy and promoting effective embryo implantation are facilitated by a healthy reproductive tract environment [172]. The vaginal microbiome of healthy, reproductive‐age women typically contains around 1 billion bacteria per gram of vaginal fluid and is characterized by low diversification, with Lactobacillus species accounting for as much as 95% of all bacteria [173]. Interestingly, the prevalence of *Lactobacillus* spp. seems to be age‐dependent and exclusively linked to reproductive age: in childhood, the majority consists of *E*. *coli* and anaerobes; during puberty, *Lactobacillus* spp. colonization begins, and after menopause, its abundance gradually decreases [102].

The vaginal microbiota undergoes constant changes, while sex hormone levels fluctuate as women age [174]. The vaginal microbiota is significantly influenced by the cyclical levels of menstrual cycle‐regulating hormones, particularly progesterone (P) and estradiol (E2), throughout a woman's reproductive years [175]. The components of the female genital tract's defensive barriers, such as the thickness of the epithelial barrier, the frequency of immune cells, the viscosity of mucus, and the vaginal microorganisms residing there, are all affected by the levels of sex hormones.

### 5.2 Body Mass

Research indicates a connection between the health of mothers' children and their pre‐pregnancy body mass index (BMI), suggesting a potential relationship between gut microbiota and reproductive health even before conception [176].

Obesity is defined as having a body mass index (BMI) of 30 or higher. Studies have associated obesity with various obstetric and gynecological issues, such as pre‐eclampsia, macrosomia, and miscarriage, and have shown an adverse correlation with factors affecting the efficacy of assisted reproduction [177]. Obesity can impact reproductive function through several mechanisms, including altered microbial states, chronic inflammation, psychological effects, reduced immunological response, and subsequent effects on the reproductive hormonal environment [178].

In comparison to lean women, overweight and obese women have a greater prevalence of bacterial vaginosis (BV), according to a cross‐sectional study conducted in the United States with over 6000 participants [179]. Increasing BMI was linked to BV in a second large cross‐sectional investigation using self‐collected vaginal swabs, although statistical analysis did not reveal this to be an independent risk factor. Pregnancy‐related BMI was linked to a higher incidence of vaginal dysbiosis during pregnancy, as evidenced by prospective research. This was seen in terms of both a lower quantity of Lactobacilli and a higher incidence of BV [180].

### 5.3 Genetic Variation

Genetic variations in the absorption, distribution, metabolism, and excretion of dietary components may explain some of the observed effects in supplementation studies. However, there has been limited research on how genetic variation influences the relationship between fertility and diet. Ultimately, the development of individualized dietary recommendations to optimize fertility may stem from a deeper understanding of an individual's genotype [181] (Table 3).

**TABLE 3:** Overview of the potential impact of genetic variation in micronutrient and macronutrient metabolism on male fertility.

Micronutrient/macronutrients	Gene and SNP	Impact	References
Retinoic acid	*BCMO1*: rs11645428	Meiosis I/II and post meiotic spermatid development	[182]
Vitamin B_12_	*FUT2*: rs602662	Sperm count, quality and motility	[183]
Vitamin C	*GSTT1: insertion or deletion*	Semen volume, concentration, sperm count, morphology, and motility	[184]
Vitamin D	*CYP2R1: rs10741657*; *GC: rs2282679*	Sperm motility and morphology; sex hormone binding globulin (SHBG)	[185]
Vitamin E	*CYP4F2: rs2108622*; *SCARB1: rs11057830*; *APOA5: rs12272004*	Acrosome reaction; sperm morphology	[186]
Folate	*MTHFR*: rs1801133	Sperm density and morphology	[187]
Choline	*CHDH: rs12676*; *PEMT: rs4646343*; *PEMT: rs7946*	Sperm motility	[188]
Betaine	*CHDH* +*432: rs12676*; *PEMT* ‐*744: rs12325817*	Spermatogenesis	[189]
Iron	*TMPRSS6: rs4820268*; *TFR2: rs7385804*; *HFE: rs1800562*; *SLC17A1: rs17342717*; *HFE: rs1799945*; *TF: rs3811647*	Spermatogenesis; sperm volume, density, motility and morphology; excess leads to oxidative DNA damage	[190]
Calcium	*GC: rs7041*; *GC: rs4588*	Sperm maturation, motility, morphology, and overall function	[191]
Omega‐3	*NOS3: rs1799983*; *FADS1: rs174547*; *FADS2: rs2727270*, *rs498793*	Sperm motility; membrane fluidity; sperm concentration	[192]
Sugar	*GLUT2: rs5400*	Sperm motility and count	[193]
Fiber	*TCF7L2: rs12255372*	Low fiber diet can lead to insulin resistance and type 2 diabetes, negatively impacting spermatogenesis, sperm maturation	[194]
Gluten	*HLA: rs2395182*, *rs7775228*, *rs2187668*, *rs4639334*, *rs7454108*, *rs4713586*	Androgen resistance; sperm morphology and motility	[195]

### 5.4 Diet

Dietary patterns serve as vital and practical nutritional tools that offer insights into a person's eating habits, which play a role in numerous complex nutritional interactions impacting overall health and well‐being. Mediterranean and Western diets have been shown to have positive and negative effects on women's reproductive health, respectively [196]. Chavarro & Co. examined how dietary habits influenced female fertility. They suggested that a diet resembling the Mediterranean diet could be considered the optimal “fertility diet.” This dietary pattern was associated with a 27% decrease in the risk of infertility and a 66% reduction in ovulatory disorders. Additionally, it was linked to a higher ratio of monounsaturated fatty acids (MUFA) to trans saturated fats, consumption of full‐fat dairy products, a lower glycemic load, and intake of vitamins [197].

Research indicates that diet influences the composition of the microbiome, genome, and epigenome, in response to an individual's genetic background, thereby impacting female reproductive health and outcomes. Various dietary factors, including polyunsaturated fatty acids (PUFA), folate, fiber, starch, and adherence to the Mediterranean diet, appear to have a positive influence on female fertility and the efficacy of assisted reproductive technologies (ART). Given the impact of diet on reproductive health, understanding the correlation between dietary intake and female fertility is crucial. For instance, trans‐saturated fatty acids may have adverse effects on female reproductive health, contributing to ovulatory disorders, reduced fecundability, and endometriosis [198].

It is essential to grasp the link between food consumption and female fertility because diet directly influences the development of long‐term metabolic disorders that can affect reproductive health, such as obesity [196].

### 5.5 Race

The vaginal microflora is crucial for successful reproduction as it plays a vital role in maintaining the host's normal physiological environment, a fact long recognized. Lactobacilli, predominant in the normal vaginal flora, especially in women of European descent, offer vaginal protection against urinary tract infections and sexually transmitted infections. Conversely, in women of African American descent, there is a different microbial composition. Alterations in the vaginal microbiota, such as reduced lactobacilli levels and increased populations of facultative and anaerobic organisms, can lead to bacterial vaginosis. This condition predisposes the host to a higher risk of bacterial infections and complications like low birth weight. During pregnancy, the vaginal microbiome changes, with a higher prevalence of *Lactobacillus* species and reduced microbial diversity. However, an altered vaginal microbiota with low levels of lactobacilli, especially during pregnancy, may lead to preterm labor and excessive inflammation [199].

### 5.6 Microbiology and Immunology

Over the past two decades, it has become evident that the gut microbiota impacts numerous metabolic and immune pathways in hosts. Conversely, the intestinal ecosystem can undergo alterations, with the composition and metabolism of intestinal bacteria being influenced by environmental conditions, lifestyle choices, and host responses [200].

Recent research indicates that certain bacteria within the gut microbiota, particularly in humans, can trigger intestinal CD4+ TH17 responses, CD4+ TH1 responses [201], or CD8+ TH1 responses [202] in the mouse gut. Segmented filamentous bacteria (SFBs) can directly and indirectly activate epithelial innate defenses by stimulating Type 3 innate lymphoid cells to produce IL‐22 and T cells to produce IL‐17. Notably, IL‐22 enhances the expression of Reg3γ in epithelial cells. SFB induces the development of inducible gut‐associated lymphoid tissue and Peyer's patches simultaneously, where they elicit specific T‐cell and adaptive IgA responses. SFB markedly increases the population of T cells expressing ROR‐γt; in C57BL/6 mice, most of these cells differentiate into TH17 cells, while a smaller fraction transforms into FOXP3 regulatory T cells. Consequently, SFB stimulates TH17 responses against itself and other commensal bacteria, contributing to the maintenance of the intestinal barrier through the induction of a state of controlled inflammation. The presence of SFB enhances the barrier effect of the microbiota [203].

The interplay between signaling molecules known as cytokines and their receptors, expressed by immune cells and host tissues, leads to inflammation in response to invading foreign agents. Cytokine networks predominantly regulate inflammation and immune responses related to infections. The immune response can be compromised as invasive microorganisms often target these cytokine mediators. For instance, *Pseudomonas aeruginosa* ( *P*. *aeruginosa* ) secretes several proteases known to be significant virulence factors. Among these are aeruginolysin (an alkaline protease) and pseudolysin (an elastase), both substrates for key proinflammatory cytokines, IL‐6 and IL‐8. This interference with leukocyte recruitment may assist *P*. *aeruginosa* in establishing an infection. Additionally, the protease AprA, capable of directly degrading epithelial‐derived IFNλ and inhibiting IFN signaling, is secreted by *P*. *aeruginosa* strains isolated from individuals with cystic fibrosis, in a manner dependent on LasR [204].

## 6 Infertility Treatment With Probiotics in Clinical Trial Study

Numerous studies have examined the effects of probiotics on infertile couples, but the results have been inconsistent (Table 4). The variations may be due to differences in probiotic species/strains, formulations, doses, intervention length, and patient circumstances. Probiotic supplements may consist of a single strain of bacteria or a combination of multiple strains or species. Research indicates that multi‐strain and/or multi‐species probiotics may, in certain instances, be more efficacious than single‐strain probiotics, as the various strains or species may synergistically enhance each other's benefits [218]. This study analyzed clinical trials that used either a single species of probiotics or a combination of several species, showing better outcomes. *Lactobacillus acidophilus* and *Lactobacillus rhamnosus* were two of the most commonly used species in treating infertility in the studies reviewed. These two bacterial species are among the best‐known probiotics globally for addressing a wide range of medical issues [219, 220]. Many researchers have studied its genetic, biological, and physiological properties. Clinical trial probiotic strains must be evaluated for safety, efficacy, and characteristics. As probiotics continue to be a valuable complementary intervention tool for modulating dysbiosis of the microbiota—which has been linked to a number of metabolic disorders and diseases—the dosage of probiotics is another critical factor to think about when studying their effects on the physiological functions in humans and other animals [221, 222]. In order to restore eubiosis, a healthy microbiota, it may be necessary to administer certain probiotic strains at certain concentrations, on the other hand, the misuse of probiotics may present risks and safety issues for individuals with compromised immune systems [223, 224]. The optimal dosage of probiotics remains unclear; however, a dose exceeding 10^6^ CFU/g (CFU/mL) is widely regarded as capable of producing highly effective outcomes. In the trials reviewed in this study, infertile patients were administered daily probiotic doses ranging from 10^8^ to 10^11^ CFU before conception. The treatment results among infertile patients indicate that the optimal suggested dosage is an average of ≥ 10^8^ CFU per g, demonstrating efficacy in inducing remission and reducing relapse and rate of complications. The outcomes align with the results of the meta‐analysis conducted by López‐Moreno and Aguilera [225].

**TABLE 4:** Investigation of the dose and mechanism of probiotics' effect on infertility treatment in several clinical trials and ongoing studies.

Author	Code trail	No. of participants (mean age ± SD)	Participants' characteristics or sample	Probiotics	Dose of probiotic (CFU)	Intervention and probiotic	Route of administration/mechanism	Outcome
Fernández et al. (2021) [205]	P050/19, Act 11/19	58 C (*n* = 14): 34.6 (33.5–35.8) RA (*n* = 21): 39.4 (38.5–40.4) INF (*n* = 23): 39.4 (38.5–40.4)	Women with repetitive abortion or infertility	*L*. *salivarius* CECT5713	9 × 10^6^	D 6 months	Oral	*L*. *salivarius* CECT5713 has proved to be a good candidate to improve reproductive success in women with reproductive failure
Thanaboonyawat et al. (2023) [206]	TCTR20190429001	340 S (*n* = 158): 35.10 ± 3.38 C (*n* = 158): 35.51 ± 3.25	Infertile women	*L*. *acidophilus* KS400	100 × 10^6^	D 6 days	Vaginal	Intravaginal lactobacilli supplementation before embryo transfer in the frozen‐thaw cycle did not improve the biochemical and clinical pregnancy rate in the general population but significantly reduced the miscarriage rate
Hariri et al. 2024 [207]	IRCT20211108053007N1	56 S (*n* = 28): 28.42 ± 6.10 C (*n* = 28): 32.75 ± 15.99	Women diagnosed with PCOS	*B*. *coagulans* (GBI‐30) *Lrhamnosus*, *L*. *helveticus*	10^11^ 10^10^ 10^10^	D 12 weeks	Oral	12‐week supplementation with synbiotics could noticeably improve the emotional, body hair, weight, infertility, and general physical health status of women with PCOS
Schenk et al. (2021) [208]	NR	80 18–40 years	Women with primary or secondary infertility	*L*. *crispatus* LBV88 *L*. *rhamnosus* LBV96 *L*. *gasseri* LBV150N *L*. *jensenii* LBV116	NR	D 4 weeks	Oral	*Lactobacillus* species could produce a temporary protective effect of the vaginal microbiota by containing or suppressing non‐beneficial bacteria, such as *U*. *parvum*
Maretti et al. (2017) [209]	2016‐1	41 S (*n* = 20): 37 (32–42) C (*n* = 21): 36 (30–43)	Male partners of infertile couples	*L*. *paracasei* 86 B21060	< 5 × 10^9^	D 6 months	Oral	Flortec constitutes a safe therapy for improving the volume of the ejaculate and the quality/quantity of spermatozoa in iOAT patients
Di Pierro et al. (2023) [210]	NCT05871242	160 S (*n* = 80): 37.53 *±* 5.12 C (*n* = 80): 37.56 *±* 4.63	Women with primary infertility	*L*. *crispatus* M247	< 20 × 10^9^	D 90 days	Oral	There is a good overall probability that women undergoing ART may benefit from oral treatment with *L*. *crispatus* M247
Jepsen et al. (2022) [211]	NCT03843112	74 S (*n* = 38): 30.6 (4.0) C (*n* = 36): 31.5 (4.5)	Women with primary infertility	*Lactobacillus gasseri* EB01 DSM14869 *Lactobacillus rhamnosus* PB01 DSM14870	> 10^8^ > 10^8^	D 10 days	Vaginal	Vaginal probiotics may not be an effective means of modulating the vaginal microbiome for clinical purposes in an infertile population. However, a spontaneous improvement rate of 34.2% over a period of one to 3 months, confirming the dynamic nature of the vaginal microbiota
Azizi‐Kutenaee et al. (2022) [212]	IR.HUMS.REC.1399.326	40 S (*n* = 20): 27.50 (7.25) C (*n* = 20): 27.50 (6.75)	Women with PCOS	*L*. *acidophilus* *B*. *bifidus* *L*. *rutri* *L*. *fermentum*	D 2 months	2 × 10^9^ 2 × 10^9^ 2 × 10^9^ 2 × 10^9^	Oral	8 weeks of administration of probiotic may improve chemical and clinical pregnancy rate, sexual function and body satisfaction in women with PCOS
Chudzicka‐Strugała et al. (2021) [213]	NCT03325023	39 S (*n* = 20): 30.8 ± 0.9 C (*n* = 19): 29.1 ± 1.3	Women with PCOS	2 strains *of* *B*. *lactis* (W51 and W52) *L*. *acidophilus* (W22) *L*. *paracasei* (W20) *L*. *plantarum* (W21) *L*. *salivarius* (W24) *L*. *lactis* (W19)	D (4 capsules) 3 months		Oral	Synbiotic supplementation potentiated effects of lifestyle modifications on weight loss and led to significant reduction of serum testosterone
Jamilian et al. (2018) [214]	IRCT20170513033941N22	S (*n* = 20): 30.8 ± 0.9 C (*n* = 19): 26.0 ± 5.3	Women with PCOS	*L*. *acidophilus* *L*. *reuteri* *L*. *fermentum* *B*. *bifidum*	8 × 10^9^	D 12 weeks	Oral	Co‐administration of probiotic and selenium for 12 weeks to women with PCOS had beneficial effects on serum total testosterone
Balaghi et al. (2020) [215]	IRCT2015062322892N1	70 18–55 years	Women with Nugent score = 4–6 and vaginal pH > 4.5	*L*. *acidophilus* *L*. *plantarum* *L*. *fermentum* *L*. *gasseri*	5 × 10^10^ 10^5^ × 10^10^ 7 × 10^9^ 2 × 10^10^	D 3 months	Oral	Lactofem oral capsule could improve the participants' Nugent scores, but caused no change in *Lactobacillus* colonization or vaginal pH
Helli et al. (2020) [216]	R.AJUMS.REC.1396.621	50 S (*n* = 25): 32.23 ± 4.11 C (*n* = 25): 33.01 ± 3.91	Infertile men	*L*. *casei* *L*. *rhamnosus L*. *bulgaricus L*. *acidophilus B*. *breve* *B*. *longum* *S*. *thermophiles*	2 × 10^11^	D 10 weeks	Oral	Probiotic supplementation in infertile men lead to a significant increase in sperm concentration and motility and a significant reduction in oxidative stress and inflammatory markers
Davari Tanha et al. (2023) [217]	IRCT20091012002576N17	94 S (*n* = 49): 33.59 ± 0.7 C (*n* = 45): 33.59 ± 0.7	Infertile women IVF treatment	*L*. *rhamnosus*	10^9^	D 2 weeks	Vaginal	A probiotic agent such as Lactovag can be useful in normalizing this environment and improving pregnancy outcomes in infertile women

Abbreviations: ART, assisted reproductive technology; C, control group; CFU, colony‐forming units; D, daily; Flortec, a probiotic associated with a prebiotic; INF group, had a history of infertility (inability to conceive) despite being the recipients of ART for at least three times, including two cycles, at least, of in vitro fertilization (IVF); iOAT, male idiopathic oligoasthenoteratospermia; PCOS, polycystic ovary syndrome; RA group, had a history of recurrent miscarriage with three or more pregnancy losses during the first 12 weeks of pregnancy.

Potential therapeutic approaches for altering the vaginal microbiota in the context of infertility in couples include targeted antimicrobial therapies, probiotics, prebiotics, fecal microbiota transplantation (FMT), vaginal microbiota transplant trials (VMT), and engineered probiotics.

Oral probiotics, such as *Lactobacillus* and *Bifidobacterium* species, have the capacity to colonize the gut and subsequently influence reproductive health [226, 227, 228]. Alternatively, they may be directly administered to the vaginal tract to restore a healthy microbial balance. These probiotics adhere to the vaginal epithelium, produce lactic acid to sustain an acidic pH, and inhibit the proliferation of pathogenic bacteria [229]. Prebiotics, which are non‐digestible dietary fibers, are essential for promoting the growth of beneficial bacteria and restoring microbial balance. They may improve sperm quality, decrease oxidative stress, and regulate the immune response in the context of male infertility [230, 231].

FMT may influence infertility by modifying the vaginal and intestinal microbiota, reducing inflammation, and improving overall reproductive health [232, 233]. Furthermore, VMTs hold potential for both clinical treatment and research regarding disease etiology, positioning them as an intriguing strategy for engineering the female sexual microbiome [234]. Engineered probiotics use genetically modified bacteria to perform specific functions, serving as an alternative to conventional pharmaceutical therapies. Treatment involving genetically modified organisms should be classified as drug therapy rather than, as with traditional probiotics, a dietary supplement [234]. Studies assessing individual bacterial strains or consortia have shown some success in treating vaginal and reproductive conditions [235].

## 7 Conclusions

The identification of a reproductive microbiota continuum has emphasized the significance of a healthy microbiome in all stages of reproduction, from gamete development to implantation to labor, involving all regions within the reproductive tract. Based on the similarities between the upper and lower genital microbiomes, the vaginal or cervical microbiome may be linked to the endometrial microbiome or even patient reproductive outcomes. The interaction of *Lactobacillus* species is critical for the normal flora of the vagina; infertility has also been linked to the presence of Gram‐negative bacteria and a lack of *Lactobacillus* in numerous studies. *G*. *vaginalis* has been identified in infertile women, and a lack of *lactobacillus*‐dominated endometrium has been linked to frequent implant failure. To completely understand the role of the microbiome, future research should focus not only on the description of microbiota but also on the interaction between bacteria, the production of biofilms, and the interaction of microorganisms with human cells.

## Author Contributions

R.G. and A.D. initiated the idea of this study. A.D., Z.E., and R.G. contributed to data collection, interpretation, and final approval of data for the work. Z.E. and M.M. developed the first and final draft of the manuscript. S.M., S.S., and F.G. developed the second draft of the manuscript. All figures and tables were designed and checked by T.D. All authors reviewed and contributed to the revisions and finalized the drafts.

## Ethics Statement

This study was approved by the Ethics Committee (Grant Number: IR.BHN.REC.1402.095) of Behbahan Faculty of Medical Sciences.

## Consent

The authors have nothing to report.

## Conflicts of Interest

The authors declare no conflicts of interest.

## Data Availability

The data that support the findings of this study are available on request from the corresponding author.
